# The complete chloroplast genome of *Ottelia acuminate* var. *crispa*, an endangered aquatic herb with extremely narrow distribution

**DOI:** 10.1080/23802359.2021.1899860

**Published:** 2021-03-22

**Authors:** Chaoyong Tu, Hao Lin, Qiang Wang, Lijun Wu, Yulian Yang, Lijuan Zou, Qinggui Wu

**Affiliations:** Ecological Security and Protection Key Laboratory of Sichuan Province, College of Life Science and Biotechnology, Mianyang Normal University, Mianyang, China

**Keywords:** *Ottelia*, plastome, endangered species, phylogenetic

## Abstract

*Ottelia acuminate* var. *crispa* is an endangered aquatic herb with extremely narrow distribution. In this study, we assembled the chloroplast genome of this species based on the second-generation high-throughput sequencing. The genome is 157,783 bp in length with a typical quadripartite structure including a large single-copy region (LSC) of 88,294 bp, a small single-copy region (SSC) of 49,379 bp, and a pair of inverted repeats (IRs) of 10,055 bp each. A total of 128 genes were annotated, including 83 protein-coding genes (PCG), 37 tRNA genes, and 8 rRNA genes. The phylogenetic tree shows that *O. acuminate* var. *crispa* has a close relationship with the genus *Elodea.* The chloroplast genome presented here provides a valuable resource to conserve this endangered species.

*Ottelia acuminate* var. *crispa* (Hand.-Mazz.) H. Li (*Ottelia*, Hydrocharitaceae) is an endangered aquatic herb that currently only exists in Lugu Lake in southwest China. As a submerged dioecious plant, water and light environment, climatic conditions, and nutrients are often a factor to it and now have been an endangered species. It was classified in the IUCN Red list as critically endangered species in 2004 (IUCN Nepal 2004). *Ottelia acuminate* var. *crispa* flowering throughout the year could be an important ornamental aquatic plant attract tourists, while its leaves are also of certain edible value. In addition, *O. acuminate* var. *crispa* could be used in monitoring water pollution and environmental protection due to its high requirements for water quality environment. However, little is known about the complete chloroplast genome (cp-genome) information of such an endangered species. In this study, we reported the cp-genome of *O. acuminate* var. *crispa*. The annotated genome has been submitted to GenBank under the accession number of MT993696 (https://www.ncbi.nlm.nih.gov/nuccore/MT993696).

We collected the fresh leaves of an *O. acuminate* var. *crispa* individual from Lugu Lake in Yunnan Province, China (100.8207E, 27.7002 N). Voucher specimen (MNU-PHO-0154) of the species was stored in the Ecological Security and Protection Key Laboratory of Sichuan Province, China. We used the DNA-secure Plant Kit (TIANGEN) to extract the total DNA from samples. The whole-genome sequencing was carried out with the HiSeq X Ten Platform (Illumina, USA). Finally, we generated about 10 G high-quality base pairs of raw data. The software fast QC (Gdula et al. 2019) and Trimmomatic (Bolger et al. 2014) were used to filter the raw data to obtain clean reads for the subsequent analysis. The software BWA v0.7.12 (Li and Durbin 2009) and SAMtools v1.3.1 (Li et al. 2009) were used to map the clean reads to the reference *Ottelia cordata* (MN056354.1) chloroplast genome. The mapped reads were extracted and then used to assemble the genome with the software NOVOPlasty v4.1 (Dierckxsens et al. 2017). The resulting contig was aligned to the reference genome by Bwa and Samtools again. We use Geneious version 8.1.4 (Kearse et al. 2012) to compare and adjust the assembled cp-genome sequence manually. Finally, we obtained a cp-genome of *O. acuminate* var. *crispa* with a size of 157,783 bp after filling the gaps with GapCloser (Luo et al. 2012). We annotated the cp-genome with Plann v1.0 (Huang and Cronk 2015) and checked the quality with Sequin v15.10 (Clark et al. 2016).

The cp-genome of *O. acuminate* var. *crispa* is with a typical quadripartite structure which consists of a large single-copy region of 88,294 bp, a small single copy region of 49,379 bp, two reverse repeat regions of 10,055 bp each and a GC content of 36.58%. Genome annotation reveals a total of 127 genes, including 84 protein-coding genes (PCGs), 35 transfer RNA (tRNA) genes, and 8 ribosomal RNA (rRNA) genes.

To infer the phylogenetic position of *O. acuminate* var. *crispa*, we reconstructed a phylogenetic tree with other 6 complete chloroplast genomes downloaded from NCBI (Figure 1) including *O. acuminate* var. *crispa*. The sequences were aligned using the software MAFFT (Katoh and Standley 2013) and the maximum likelihood analysis worked on software RAxML v8.2.9 (Stamatakis 2014) setting GTRGAMMA as the best model and 1000 bootstrap tests. The phylogenetic tree demonstrates that *O. acuminate* var. *crispa* is closely related to genus *Elodea* with strong support (Figure 1).

**Figure 1.**
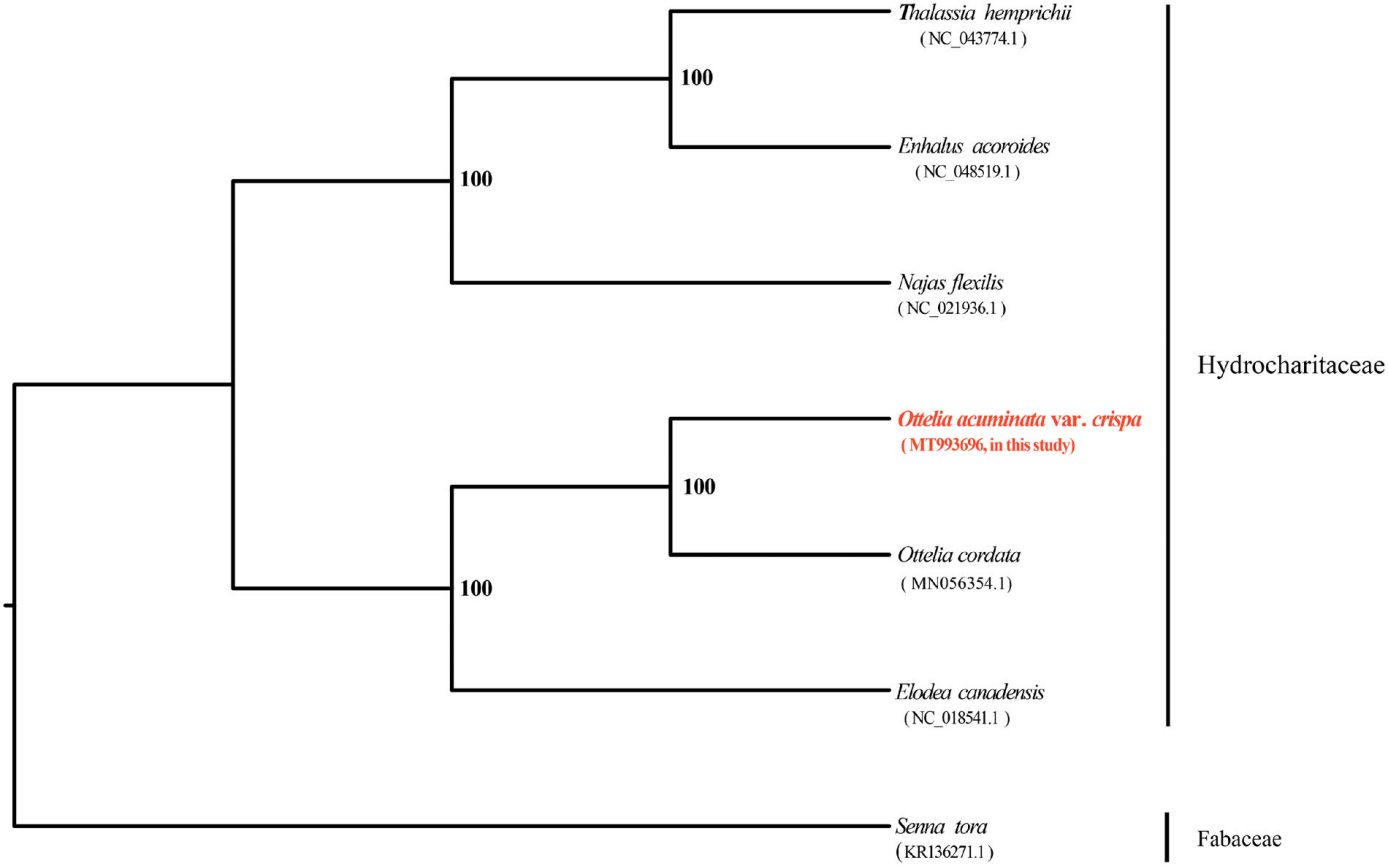
Maximum likelihood phylogenetic tree based on the cp-genome sequences of *Ottelia acuminate* var. *crispa* and other 6 species. Numbers in the nodes are the bootstrap values from 1000 replicates. The numbers in brackets are the NCBI accession number of each species.

In summary, the complete chloroplast genomic of *O. acuminate* var. *crispa* will not only provide valuable informative resource to facilitate the identification, conservation, and utilization of this extremely endangered species but also provide insightful information to further research on phylogenetic study of Hydrocharitaceae.

## Data Availability

The annotated genome has been submitted to GenBank under the accession number of MT993696 (https://www.ncbi.nlm.nih.gov/nuccore/MT993696). The voucher specimen of the species is free accessible at Ecological Security and Protection Key Laboratory of Sichuan Province, China with the No. MNU-PHO-0154.
